# Utilization of high-fidelity simulation to address challenges with the basic science immunology education of preclinical medical students

**DOI:** 10.1186/s12909-019-1786-5

**Published:** 2019-09-14

**Authors:** Marie Cavuoto Petrizzo, Maria-Louise Barilla-LaBarca, Youn Seon Lim, Artemio M. Jongco, Michael Cassara, James Anglim, Joel N.H. Stern

**Affiliations:** 1 Donald and Barbara Zucker School of Medicine at Hofstra/Northwell, 500 Hofstra Blvd., Hempstead, NY USA; 2 Northwell Health Division of Allergy and Immunology, 865 Northern Blvd., Great Neck, NY USA; 3 Northwell Health Patient Safety Institute, 1979 Marcus Avenue, New Hyde Park, NY USA

**Keywords:** Simulation, Basic science, Immunology, Preclinical medical students, Integration

## Abstract

**Background:**

Immune function and dysfunction are highly complex basic science concepts introduced in the preclinical medical school curriculum. A challenge for early learners is connecting the intricate details and concepts in immunology with clinical manifestations. This impedes relevance and applicability. The impetus in medical education reform is promoting consolidation of basic science and clinical medicine during the first two years of medical school. Simulation is an innovation now widely employed in medical schools to enhance clinical learning. Its use in basic science curriculums is largely deficient. The authors piloted simulation as a novel curricular approach to enhance fundamental immunology knowledge and clinical integration.

**Methods:**

The authors introduced a Primary Immunodeficiency Disease (PIDD) simulation during a basic science immunology course for second-year medical students at the Zucker School of Medicine at Hofstra/Northwell. The simulation tasked small groups of students with evaluating, diagnosing and managing an infant with previously undiagnosed immunodeficiency. Joint facilitation by clinical and science faculty during terminal debriefings engaged students in Socratic discussion. Debriefing aimed to immerse basic science content in the context of the clinical case. Students completed a post-simulation Likert survey, assessing utility in reinforcing clinical reasoning, integration of basic science and clinical immunology, enhanced knowledge and understanding of immunodeficiency, and enhanced learning. A summative Immunodeficiency Objective Structured Clinical Examination (OSCE) question was created by faculty to assess students’ recognition of a PIDD and clinical reasoning.

**Results:**

The simulation was well received by students with > 90% endorsing each of the objectives on the post-simulation survey. The authors also determined a statistically significant score variance on the summative OSCE question. Higher scores were achieved by the cohort of students completing the OSCE post-simulation versus the cohort completing the OSCE pre-simulation.

**Conclusions:**

The innovative use of simulation in a highly complex basic science immunology course provides relevance and consolidation for preclinical learners. Additional data will be collected to continuously assess application of concepts and proficiency stemming from this novel curricular intervention. The authors advocate the initiation and/or expansion of simulation in non-clinical basic science courses such as immunology to bridge the gap between theory and practice.

## Background

The first two years of undergraduate medical education have traditionally been defined by Flexner’s model, grounded in foundational basic science coursework [1]. It was not until the 1980s that Bloom reported the dysfunctions of the existing medical education system, one of which purported “the primacy of scientific knowledge” without practical application [2]. Further impetus for change was driven by the recommendations of Cooke et.al in their 2010 report “Educating Physicians: A call for Reform of Medical School and Residency” [3]. The authors advocated an assimilated approach between basic, clinical and social science education and a connection of formal knowledge with clinical experience [3]. Medical schools responded to this call of action recognizing the need to incorporate clinical application within the basic science curriculum [4]. This integrative approach recognizes the value of basic science knowledge in providing causal relationships and explanatory models for clinical occurrences [5].

Simulation emerged in medical education as a method to integrate knowledge and practice. Full environment high fidelity mannequin simulation is now widely employed by medical schools. Okuda et al. outlined the multiple domains full environment simulation impacts medical education [6]. The authors note it serves to enhance clinical skills instruction and affords learners the ability to train on new technologies without risk of harm to patients [6]. Simulation endorses teamwork and the development and practice of communication skills [6]. In addition, simulation promotes the consolidation of knowledge [6].

An Association for Medical Education in Europe (AMEE) guide published 2013 presented an updated model of learning theories combining many of the conventional theories [7]. Simulation is compatible with this multi-theory model. Learning is structured upon base knowledge [7], which in the case of simulation, may be derived from readings, didactic sessions and problem/case-based learning sessions. The dissonance phase of this model [7] is entered when students engage in the simulation experience. Student knowledge is challenged, deficits in knowledge are exposed, and new possibilities are introduced. During the refinement phase of the model [7], students reflect on the experience during debriefing and discuss new information, concepts, and explanations. The organization phase [7] also occurs during the debriefing process as students incorporate new material and explanations, assess these notions, and reform their ideas. Debrief facilitators, who are content experts, support the students in the role. Student articulation of knowledge occurs in the feedback phase [7], with debrief allowing for dialog amongst students and faculty. Finally, during the consolidation phase of this model [7], learners will be able to reflect on their knowledge acquisition, the learning process and the construction of the “big picture” [7].

While simulation is utilized for reinforcing clinical skills, teamwork and communication skills, and physical diagnosis [6, 8], there are noticeably few reports of its use in preclinical basic science education [9–11]. When utilized for basic science knowledge consolidation, it is predominately purposed for anatomy, pharmacology and physiology teaching [9, 12–21]. A survey by the Association of American Medical Colleges (AAMC) found that less than 10% of US medical schools surveyed used simulation for preclinical immunology content [9]. We recognize this underutilization as a missed educational opportunity to impart integration and consolidation.

Medical students view immunology as an “esoteric subject” [22]. Students often struggle with the complexity of immunology as a science and fail to integrate “theoretical immunology and its practical application” [22]. This lack of assimilation between basic immunology concepts and clinical manifestations results in students largely memorizing principles, pathways and mechanisms. Consequences may manifest as deficits in retention and conceptual knowledge. Simulation appeals to millennial learners due to its interactive and “hands-on” design, in contrast to didactic sessions [23]. The experience of simulation may fill the gap in immunology teaching and allow for both contextualization and relevance for the millennial learner [21].

Students in the second year of medical school at the Donald and Barbara Zucker School of Medicine at Hofstra/Northwell participate in a basic science immunology course. Due to the heavy basic science coursework requirement, only 2 h of curricular time is devoted specifically to an overview of primary immunodeficiency diseases (PIDD). The format is a large class didactic session. An additional 4 h is spent in hybrid problem/case-based learning sessions constructed around PIDD vignettes. We recognize the study of PIDD provides an opportune venue to bridge understanding of aspects of basic and clinical science during this course. Comprehension of normal immune system function is fundamental to recognizing and evaluating clinical outcomes of immune system dysfunction.

A challenge for our early learners has been connecting the intricate details and concepts in immunology with clinical manifestations. Student weaknesses include linking various immunodeficiencies with specific arms of the immune system, categorizing corresponding infections within each arm, and understanding why specific laboratory evaluations would be most pertinent to utilize. We predicted that use of simulation would link “theory and practice,” enhancing students’ proficiency in both basic science and clinical immunology.

In the Fall of 2018 we piloted a novel PIDD simulation case for second year medical students at the conclusion of their 8-week foundational basic science immunology/rheumatology course. The simulation case featured Severe Combined Immunodeficiency Disease (SCID) to highlight immune cell development and cellular and humoral immune system dysfunction through a clinical lens. To our knowledge, there are no published reports of the utility of simulation to educate medical students in a preclinical basic science immunology course.

## Methods

### Participants and simulation design

Hofstra University’s Institutional Review Board (IRB) approved the research conducted in this study under Exempt Review procedures on October 22, 2018. Exemption status was granted per the US Code of Federal Regulations 45CFR46.101(b)(2) [24].

This pilot PIDD simulation included students enrolled in their second year of medical school at the Donald and Barbara Zucker School of Medicine at Hofstra/Northwell during the Fall of the 2018–2019 academic year. The students were equipped to partake in simulation because of our highly integrated curriculum. Students participated in a physical examination course, communications/history taking course and clinical reasoning course during their first year of medical school [25, 26]. All medical students were Emergency Medical Technician (EMT) certified during the first year of medical school and longitudinally participated in EMT shifts during year one [27]. Students also participated in multi-specialty ambulatory clinical experiences year one and continuing into year two [25, 26]. These afforded students the ability to provide initial patient management.

The students participated in this required formative simulation exercise at the end of their eight-week foundational immunology/rheumatology course. Students previously participated in 8 simulation cases during their first year of medical school and were thus familiar with the format of the simulation sessions. Simulation occurred at Northwell Health’s Center for Learning and Integration Patient Safety Institute (PSI). PSI is accredited by the Society of Simulation in Healthcare (SSH) and houses 20 simulation rooms.

The class of students (*N* = 102) was randomly divided into 2 cohorts by our curriculum support team, with each cohort participating in the simulation during one of the two days it was administered in sessions. The simulation was scheduled as an end-of-course formative curricular exercise for the students and thus no specific pre-work or pre-reading material was required. Groups of 6 students were randomly assigned to each of the simulation rooms. Three students took on the role of active participants for the case and were tasked with taking a relevant history, employing clinical reasoning, performing a hypothesis-driven physical exam and providing initial patient management. The remaining three students actively observed in the simulation room and were expected to equally participate in the debrief. Roles were determined by the students just prior to the session. Immediately following this first case, the students were presented with an unrelated case. Those students who observed the first case actively participated in the second case.

Two standardized patient actors were employed for all groups of students over the two days simulation was run and acted as the mannequin patient’s parent. Actors received a detailed script for the case for their review and memorization prior to the sessions. The actors received training by certified SSH faculty at PSI and met with the corresponding author prior to the sessions to answer questions or concerns.

Simulation rooms were designed to replicate an emergency department room. Upon entering the room, students encountered a high-fidelity simulated infant mannequin and a “standardized parent”. In the play of this case, the infant mannequin presented to the emergency department with a history of fever, cough and inconsolability. A thorough interview of the standardized parent would divulge the infant’s history of recurrent infections (i.e., otitis media, pneumonia, rashes), chronic diarrhea, failure to thrive, and a maternal uncle’s death in infancy. Examination of the mannequin revealed an irritable, grunting seven-month old male child with evidence of fever, shock, hypoxia, thrush and fungal dermatitis. Requested laboratory studies were significant for severe lymphopenia. A chest roentgenogram image and report were consistent with *Pneumocystis jiroveci* pneumonia. The simulation coursed for 15 min and ideally concluded with students recognizing Severe Combined Immunodeficiency Disease and providing basic initial patient care.

Students were supported through the case if needed. A confederate in the room took on the role of a nurse and was able to prompt students if challenged. Additionally, students were able to call for a specialist “consultation” via telephone. Faculty observing the simulation via a one-way mirror could field these calls and provide assistance to struggling students.

Students were debriefed by a basic science faculty member (Ph.D. with expertise in immunology and/or microbiology) paired with an American Board of Allergy and Immunology certified physician (M.D.) immediately after completion of the simulation exercise. The total of 3 faculty pairs observed the simulations and facilitated simultaneous debriefs sessions. The same pairs of faculty were utilized over the two days. This faculty combination attempted to elicit the critical, shared teaching approach to integrative learning [28]. Faculty members participated in a required six-hour training course on advocacy and inquiry debrief techniques [26, 29]. SSH certified simulation experts were on-hand and directly observed the simulations and debriefing process. Each facilitator also received a faculty guide highlighting goals, learning objectives, and prompted discussion points in order to standardize the debriefing encounters (Additional file 1). A one-hour case specific faculty development meeting occurred immediately prior to each of the simulation sessions. This provided additional support for faculty, and enabled discussion and review of debriefing aims.

A Socratic approach to facilitation was employed by all faculty. Faculty engaged students in an active learning dialogue of normal immunologic development and immunologic signaling pathways. Students were asked to correlate immunologic developmental and pathway aberrations with the occurrences of specific immunodeficiencies. These immunodeficiencies included X-linked agammaglobulinemia (XLA), hypogammaglobulinemia, Common Variable Immunodeficiency Disease (CVID), DiGeorge Syndrome, Severe Combined Immunodeficiency Disease, Bare Lymphocyte Syndromes, and Hyper IgM. Faculty prompted higher order conversations comparing and contrasting these diseases based on basic science knowledge and clinical appraisal. Faculty also facilitated students in the interpretation of diagnostic tests and therapeutic management. Debrief encounters lasted approximately 25–30 min for this case.

### Evaluation

We administered a standardized evaluation form (Additional file 2) to all second-year medical students simultaneously at the end of the immunology/rheumatology final exam period, approximately one week after participation in simulation. This timing coincided with our standard end of course evaluation distribution, and so as not to burden students during their examination period. Scores from a summative Objective Structured Clinical Exam (OSCE) and written final exam were not available to students at the time the evaluations were distributed and collected and did not influence the evaluation.

Students were asked to voluntarily and anonymously complete the form. The evaluation consisted of a four item, 5-point Likert as well as an area for open comments/suggestions. The items assessed the simulation learning objectives. These included integration of basic science and clinical immunology, enhanced knowledge and understanding of PIDD, reinforcement of clinical reasoning skills, and enhanced learning via faculty debrief.

We analyzed objective data by comparing scores on a summative end-of-course, faculty-created OSCE post-encounter question. During the OSCE, students were tasked with taking a history and performing a hypothesis-driven physical exam on a young-middle aged adult female standardized patient. The patient presented to an outpatient medical office with a history of recurrent sinopulmonary infections, chronic diarrhea, and a recent diagnosis of bronchiectasis. She complained of acute “sinus” symptoms and on exam demonstrated otitis media with effusion, rhonchorus breath sounds, fingernail clubbing, and a petechial skin rash illustrative of idiopathic thrombocytopenic purpura. Suspected underlying diagnosis was CVID. The OSCE post-encounter question required students make a diagnosis, justify reasoning, and provide an alternative reasonable diagnosis. A formal grading rubric was used to assign scores. Points were allocated for correctly identifying the underlying disease, providing supporting evidence for this diagnosis based on history and physical exam findings, providing a reasonable alternative diagnosis, and depth of explanation. All responses were graded by a single faculty member for consistency. The grader was blinded to student identifiers as well as date the OSCE was taken. Both the OSCE and simulation occurred during the same week. The OSCE was administered over a 3-day period per the predetermined Zucker School of Medicine exam week schedule based on the availability of faculty and standardized patients. Students were randomly assigned by curriculum support to one of the three OSCE sessions. One OSCE session occurred before simulation while the other two occurred after simulation. We determined variance in scores between the cohort of students who completed the OSCE before participating in simulation, and the cohort who completed the OSCE after participating in simulation.

This study occurred within the framework of the immunology/rheumatology course. Neither simulation or the OSCE scenario were created solely for research purposes. The content of both cases was based on the course goals and objectives and timeline subject to the predetermined course examination schedule.

### Statistical analysis

The Likert scale plot was performed for the standardized evaluation question responses. The descriptive analysis of the responses is presented as percentage frequency of students strongly agreeing, neutral, disagreeing, or strongly disagreeing with each of the four proposed objectives.

A two-tailed independent-sample t-test was used for comparison between the pre-simulation and post-simulation cohorts’ OSCE post-encounter question scores. The data are presented as mean scores (Max = 12) for each cohort and standard deviations.

The statistical software R was used for data analyses (version 3.5.0; The R Foundation for Statistical Computing, KS, USA).

The data generated and analyzed during this study are included in this article’s Additional files 3 and 4.

## Results

**Fig. 1:**
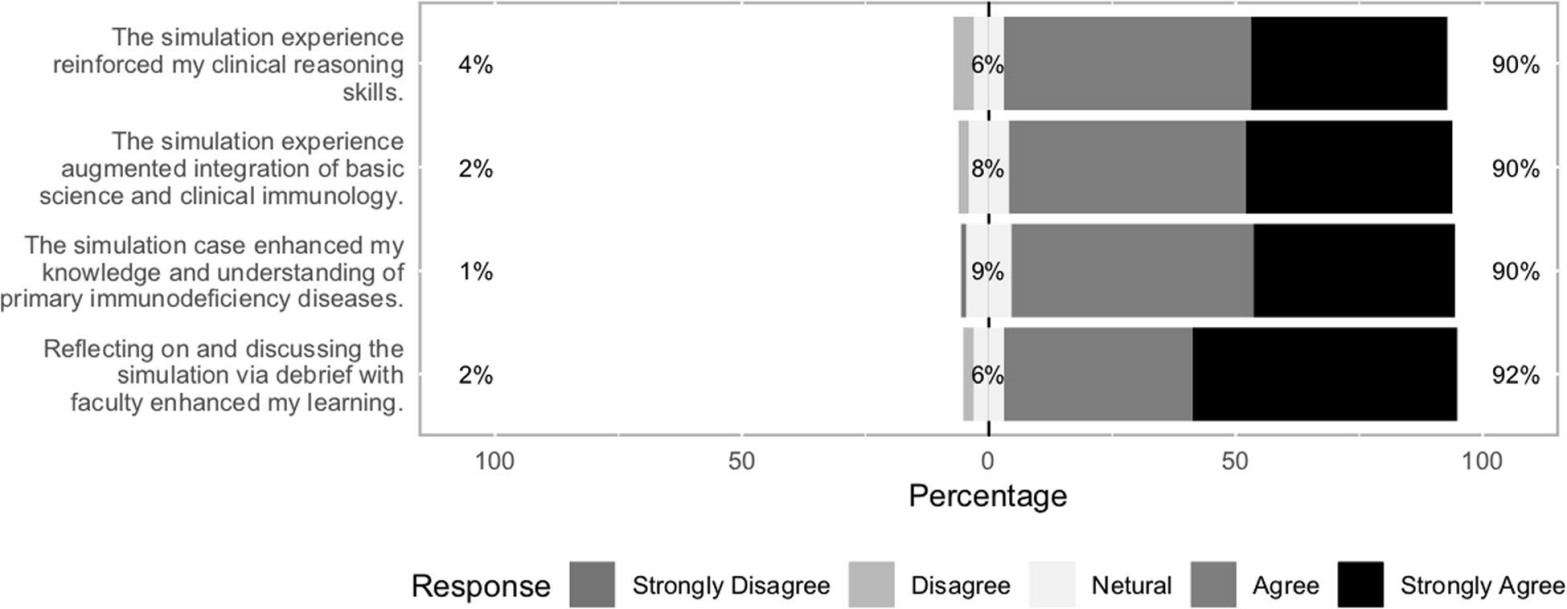
Likert scale plot for simulation session evaluation results from students (*N* = 98). Each bar represents one question. The white colored area counts neutral responses. Grayish colored bars on the left indicate disagree or strongly disagree responses, and the blackish colored bars agree or strongly agree responses as shown in the legend. The percentage of students responding positively (i.e., agree and strongly agree) to the individual queried items ranged from 90% (*n* = 88) to 92% (*n* = 90)

Ninety-six percent of students (*N* = 98) participating in the mandatory simulation experience completed the Likert evaluation and provided comments. Results reflected the clear majority of students endorsing the simulation’s success. The percentage of students responding positively to the individual queried items ranged from 90% (*n* = 88) to 92% (*n* = 90) (Fig. 1).

Representative comments from the student evaluation are mapped below to either curricular model of instruction or suitability to the discipline of immunology.

### Curricular model of instruction

“Would like more simulations during the courses”.

“Was a good experience and debriefing was very helpful. Was not so clear earlier in the course how important the immunodeficiencies were so this was helpful”.

“The discussion afterward was very high yield”.

“As a learner, I would take a well-organized flowchart/outline/PowerPoint by itself over discussion without visual aids, regardless of the quality of discussion”.

“This case was a very good presentation of an immunodeficiency, and it launched a good discussion following the simulation”

“The added challenge of communicating with the patient's family was beneficial”.

“Nice way to consolidate material”.

### Suitability to discipline of immunology

“I thought this was a great simulation case because it introduced us to the challenges of caring for a baby in an emergency situation, required clinical reasoning skills, and enhanced my understanding of primary immunodeficiencies (a relatively challenging topic) especially in the debrief”.

“Appreciated the review of clinical manifestations of immunodeficiencies, in particular the difference between B-cell and T-cell deficiencies”.

“Walking through the different immunodeficiencies was great”.

“The debrief discussion with faculty was very helpful for reviewing the presentations of various PIDDs”.

“The simulation helped me to clarify the SCIDs and their classification, which I think is a good proxy for understanding the different aspects of adaptive immunity. For example, I had thought of Omenn syndrome and type II BLS as being very similar, but SIM really elucidated the difference for me”.

“I thought this was a really good case to bring to light the reality of primary immunodeficiency disease”.

“I wish perhaps we would have known some more acute interventions to do to stabilize the child, but maybe that was not the exact point of the simulation”.

“We arrived at SCID but did not know how to proceed after that”.

“It was hard to know what to do for the patient in the SIM itself since we never learned any acute treatments for infections yet”.

The majority of qualitative responses reflected the “high-yield” nature of the debrief portion of the simulation experience. Students valued working through a differential diagnosis and correlating basic science concepts with disease presentation and manifestations. Only one student noted a preference for a more traditional method of teaching over debrief discussion. A small number of students referenced uncertainty with the initial medical management of a patient with PIDD.

**Fig. 2:**
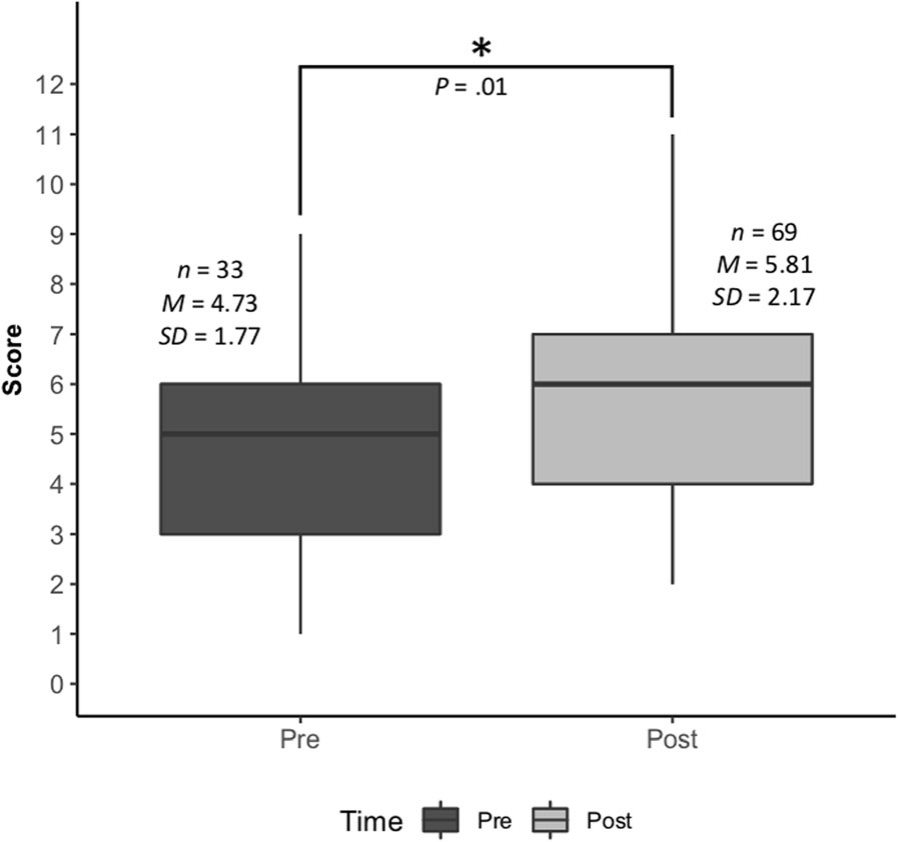
Comparison of pre- and post-OSCE PIDD question score (Max = 12). The results of a two-tailed independent-sample t-test showed a modest increase in total score when tested after the simulation experience (*t* (100) = − 2.45, *P* = 0 .01)

Data collected from the OSCE post-encounter response scores provided objective assessment of the simulation. Approximately one-third of the class participated in simulation prior to completing the OSCE, while the remainder of the class completed the OSCE after participating in the simulation case. The OSCE post-encounter response scores of pre and post-simulation student cohorts were compared. There was a modest, statistically significant improvement in overall mean question scores for students taking the OSCE after having completed simulation, as compared to those who had not yet participated in the simulation (*P* = 0.01) (Fig. 2).

It can be implied that participation in the simulation activity improved recognition of a PIDD resulting from better understanding and application of immunology concepts.

## Discussion

Results of previous studies examining the educational efficacy of simulation compared to more conventional teaching modalities has been mixed [30]. Several studies have shown improved transfer of knowledge in simulation cohorts [31–34] while others have shown no significant difference [14, 35, 36]. Utilization of simulation in the preclinical foundational immunology curriculum is an innovative method of integrating the intricacies of basic immunology with disease outcomes. It can provide applicability and relevance to a highly complex discipline. Students endorse the use of this novel approach within the preclinical immunology curriculum. They cite enhanced integration of science and clinical material in this platform. Objective data support improvement in the application of basic immunology concepts in clinical reasoning.

Limitations of this study include the smaller sample size of pre-simulation OSCE takers and the additional 24–48 h of study time available to students taking the OSCE later in the week. The uneven distribution of students in the pre- and post-simulation OSCE cohorts was due to the student scheduling and could not be modified for our study. Each of these may have impacted the mean post-encounter response scores on the OSCE. We will continue to run this simulation session for future classes of students. This will increase the sample size and power of the study. In addition, a common challenge cited by the learners referenced a lack of knowledge in managing a PIDD patient in an acute care setting. We acknowledge that the students’ lack of clinical expertise and uncertainty may have negatively impacted post-simulation evaluation responses. We also recognize the atypical nature of performing an immune evaluation in an emergency department setting. We will now allot time during our PIDD didactic session to include the initial approach to a patient with PIDD.

This simulation will now be utilized as a standard educational activity in the foundational immunology curriculum at our institution. It can be employed for both teaching as well as assessment. We plan to survey the next cohort of students and comparatively assess their satisfaction with didactic sessions, problem-based learning sessions and simulation sessions in immunology. We will continue to collect data from student evaluations and summative OSCE encounters over the next several years. This data will be used to identify areas of achievement as well as areas requiring reinforcement in the current immunology curriculum. Such information will be used to enrich the curriculum for future students. We also plan to assess application of concepts post-simulation. We will compare our student performance on the immunology sections of National Board of Medical Examiners (NBME®^)^ exams before and after the introduction of simulation in our curriculum.

## Conclusions

One of the central goals of medical education reform is to integrate basic and clinical science early in the medical school trajectory. Simulation can bridge the gap between theory and practice and enhance proficiency. Simulation use in a basic science immunology course quantitively and qualitatively enhanced student learning. We encourage medical educators to initiate the novel use of simulation within their immunology basic science medical education curriculum.

## Supplementary information

Additional file 1: Simulation Faculty Debrief Guide. Faculty facilitation guide used during simulation debrief (DOCX 22 kb)

Additional file 2: Simulation Session Likert Evaluation. Post-simulation survey template administered to students for simulation assessment. (PDF 90 kb)

Additional file 3: Simulation Likert Evaluation Raw Data. Raw data for Likert Post Simulation Evaluation Data is organized in columns corresponding to each of the 4 items assessed and scaled 1–5. (XLSX 14 kb)

Additional file 4: OSCE Raw Scores for Pre and Post Simulation Cohorts. Raw scores on the OSCE question Data is arranged by pre-simulation and post-simulation cohorts of students. Scores may range from 1 to 12 points. (XLSX 11 kb)

## Data Availability

All data generated or analyzed during this study are included in this article’s Additional files 3 and 4.

## Abbreviations

AAMC: Association of American Medical Colleges
AMME: Association for Medical Education in Europe
CVID: Common Variable Immunodeficiency
EMT: Emergency Medical Technician
NBME®: National Board of Medical Examiners
OSCE: Objective Structured Clinical Examination
PIDD: Primary Immunodeficiency Disease
PSI: Northwell Health’s Center for Learning and Integration Patient Safety Institute
SCID: Severe Combined Immunodeficiency Disease
SSH: Society of Simulation in Healthcare
XLA: X-linked Agammaglobulinemia
