# Supplementary material for: Utilization of high-fidelity simulation to address challenges with the basic science immunology education of preclinical medical students
Source: BMC Med Educ. 2019 Sep 14;19:352. doi: 10.1186/s12909-019-1786-5 (PMC6744639; doi:10.1186/s12909-019-1786-5)
Supplement: Supplementary file 1 — Simulation Faculty Debrief Guide. Faculty facilitation guide used during simulation debrief (DOCX 22 kb) [file 12909_2019_1786_MOESM1_ESM.docx]

**Additional File 1**

**SIMULATION FACULTY DEBRIEF GUIDE**

Case synopsis: Michael Peak is a 7-month old male infant with history of recurrent infections, chronic rash, diarrhea and failure to thrive, brought to the emergency room for fever and respiratory distress. The patient’s underlying disease is **Severe Combined Immunodeficiency (SCID),** now with acute *Pneumocystis jiroveci* pneumonia.

At the completion of this small group session, participants will meet the following **goals**:

1. Recognize the warning signs of a primary immunodeficiency in a pediatric patient.
2. Understand basic immunology pathways and how aberrations causally lead to disease.
3. Understand the urgency in making a diagnosis of primary immunodeficiency disease in order to immediately begin appropriate treatment(s) and avoid life-threatening complications.

At the completion of this small group session, participants will meet the following **learning objectives**:

1. Detail how precise immune cell development abnormalities result in specific primary immunodeficiency diseases.
2. Develop differential diagnoses of primary immunodeficiency diseases based on history and physical exam findings. Compare and contrast the clinical manifestations of these diseases.
3. Identify and rationalize which laboratory tests should be performed on a patient with a suspected primary immunodeficiency disease.
4. Explain the rationale for the initial management of an infant with suspected SCID.
5. Identify and discuss the treatment and outcomes of a patient with SCID.

**Suggested Prereading for Faculty**

Abbas AK, Lichtman AHH, Pillai S. Cellular and Molecular Immunology. Elsevier; 2017. Please read Chapter 21, 459- STOP at Section "Secondary (Acquired) Immunodeficiencies".

Puck JM. Laboratory technology for population-based screening for severe combined immunodeficiency in neonates: The winner is T-cell receptor excision circles. Journal of Allergy and Clinical Immunology 2012;129:607–16. doi:10.1016/j.jaci.2012.01.032.

Turvey SE, Bonilla FA, Junker AK. Primary immunodeficiency diseases: a practical guide for clinicians. Postgraduate Medical Journal 2009;85:660–6. doi:10.1136/pgmj.2009.080630.

Vogel BH, Bonagura V, Weinberg GA, Ballow M, Isabelle J, Diantonio L, et al. Newborn Screening for SCID in New York State: Experience from the First Two Years. Journal of Clinical Immunology 2014;34:289–303. doi:10.1007/s10875-014-0006-7.

Faculty debrief questions:

1. **What key historical and examination components were pertinent to your clinical reasoning in this case**?
2. **Discuss the differential diagnosis of immunodeficiency diseases for this patient? Discussion may include:**
   1. X-linked agammaglobulinemia (XLA)
   2. Hypogammaglobulinemia
   3. Common Variable Immunodeficiency (CVID)
   4. DiGeorge syndrome
   5. Severe Combined Immunodeficiency (SCID)
   6. Bare Lymphocyte Type 1 and Type II Syndromes (MHC I and MHC II Deficiencies)
   7. Hyper IgM Syndrome
3. **What are the contributions of T and B cells in each of the immunodeficiencies in your differential diagnosis?**
4. **What specific laboratory evaluations might be used to diagnose a suspected immunodeficiency? Discuss the utility of these tests.**

Quantitative tests:

**WBC count** / **total absolute lymphocyte count**

**B and T cell enumeration (lymphocyte subset analysis)**

**Serum Immunoglobulin levels (IgG, IgA, IgM, IgE)**

**T-cell Receptor Excision Circle assay (TREC assay)**

Functional tests:

**Antibody titers**

**Lymphoproliferative (Mitogen) assays**

Additional

**HIV testing**

1. **Predict the results of laboratory evaluations for the immunodeficiencies in your differential diagnosis.**
2. **Discuss the initial management of, and treatment for SCID patient?**

Include in discussion:

- - - treatment of infection
    - reverse isolation
    - irradiated blood products if transfusion required
    - withholding of vaccines
    - bone marrow transplant
    - enzyme replacement therapy
