# Supplementary material for: Utilization of high-fidelity simulation to address challenges with the basic science immunology education of preclinical medical students
Source: BMC Med Educ. 2019 Sep 14;19:352. doi: 10.1186/s12909-019-1786-5 (PMC6744639; doi:10.1186/s12909-019-1786-5)
Supplement: Supplementary file 2 — Simulation Session Likert Evaluation. Post-simulation survey template administered to students for simulation assessment. (PDF 90 kb) [file 12909_2019_1786_MOESM2_ESM.pdf]

Additional File 2

Simulation Session Evaluation

Please answer the following questions regarding the simulation sessions referenced below. The results will provide feedback to the School of Medicine for updating its curriculum and may be used in medical education research. By filling out this section you consent to the use of this data for medical educational purposes and publications.

	Strongly Disagree	Disagree	Neither Agree nor Disagree	Agree	Strongly Agree
	1	2	3	4	5
The simulation experience augmented integration of basic science and clinical immunology.					
The simulation case enhanced my knowledge and understanding of primary immunodeficiency diseases.					
The simulation experience reinforced my clinical reasoning skills					
Reflecting on and discussing the simulation via debrief with faculty enhanced my learning					

Please provide any additional comments below.
